# The Identity and Mineral Composition of Natural, Plant-Derived and Artificial Sweeteners

**DOI:** 10.3390/molecules28186618

**Published:** 2023-09-14

**Authors:** Anna Leśniewicz, Maja Wełna, Anna Szymczycha-Madeja, Paweł Pohl

**Affiliations:** Analytical Chemistry and Chemical Metallurgy Division, Faculty of Chemistry, Wroclaw University of Technology, Wybrzeze St. Wyspianskiego 27, 50-370 Wroclaw, Poland; maja.welna@pwr.edu.pl (M.W.); anna.szymczycha-madeja@pwr.edu.pl (A.S.-M.); pawel.pohl@pwr.edu.pl (P.P.)

**Keywords:** natural, plant-derived and artificial food sweeteners, phase analysis, XRD, mineral composition, multielement analysis, ICP-OES

## Abstract

The qualitative X-ray phase analysis of natural and artificial food sweeteners was applied to trace the authenticity of such food additives. The mineral composition of different sweeteners commonly added to foods was studied to estimate their mineral profiles and assess the risk related to the toxic elements intake caused by sweetener consumption. The concentration of twenty elements (Ag, Al, B, Ba, Bi, Ca, Cd, Co, Cr, Cu, Fe, Mg, Mn, Mo, Ni, Pb, Sr, Ti, V, and Zn) was measured using the inductively coupled plasma–optical emission spectroscopy (ICP-OES) method after the representative samples were wet-digested with a concentrated nitric acid and hydrogen peroxide mixture in a closed-vessel microwave-assisted system. Differences between the mineral compositions of the examined sweeteners were statistically evaluated and discussed. The relationships between the concentrations of the elements determined in the analyzed sweeteners were also investigated. The successful application of the X-ray powder diffraction method proved the identity of all investigated sweeteners; all the analyzed products contained the expected sweetening agent. The results of the quantification of all the elements in the examined sweeteners indicated that these products cannot be considered nutritionally dense. Hence, the presence of toxic elements like Cd, Cr, Ni, and Pb distinctly indicates the need to test such products to guarantee their quality and ensure consumer safety.

## 1. Introduction

Sweeteners are natural and artificial food additives that provide a sweet taste in food and beverages.

In prehistoric times, the sweet taste of ripe fruits and vegetables guaranteed the hunting man the right dose of energy, and thus survival. People like sweet taste. Furthermore, they have continuous access to unlimited—compared to historical times—resources of natural sweeteners, e.g., honey or maple syrup. The most popular way to sweeten food products is to add food sugar, i.e., sucrose, in the form of cane or beet sugar. Sweetening properties have also been observed in substances such as honey, maple, or agave syrup. Unfortunately, all-natural sweeteners have high caloric value and glycemic index; therefore, if consumed in excess, especially as added sugars, they are recognized as a cause of weight gain and obesity, tooth decay inflammation, and oxidative stress [1]. In particular cases (such as type 1 diabetes (T1D) and type 2 diabetes (T2D)), the consumption of natural sweeteners higher than the recommended 10% of daily total calories can also cause the inability to maintain blood glucose levels at the appropriate level [2]. 

The category of artificial sweeteners includes aspartame, saccharin, acesulfame K, sodium cyclamate, and sucralose. Synthetic sweeteners are popular among consumers due to their limited or even lack of calorie content, which is why they can be found in many kinds of foods and drinks labeled sugar-free or diet. Artificial sweeteners are safe for healthy people in limited amounts. The replacement of added sugars with sugar substitutes could lower the risk of tooth decay and cavities and could help manage the blood glucose level and weight in the short term. However, there is no clear answer to the question of what the long-term health effects of sugar substitute use can be. This is why increasingly conscious consumers are looking for healthy alternatives to sugar and artificial sweeteners, paying attention to sweeteners of natural origin, such as xylitol or erythritol.

Most of the recent scientific research on sweeteners focuses on the impact of consuming food products containing sweeteners, especially non-nutritive ones, which are recommended for people with diets aimed at weight reduction or blood glucose regulation, on human health and dietary preferences. For example, the effect of low, regular, and high dietary sweetness exposure on the preference of Dutch adults for sweet foods and beverages was studied [3]. Intensive research was conducted on the relationship between the consumption of products containing non-nutritive sweeteners and body weight [4,5]. Also, the impact of low-calorie sweetener consumption among adults with type 1 diabetes (T1D) and type 2 diabetes (T2D) on the quality of life (glucose control, hypertension, or any other complication) and biochemical parameters related to metabolic functions was studied [6,7]. Another direction of research related to sweeteners encompasses studies verifying the possibility of using plant-derived and artificial sweeteners as substitutes for sugar and other natural sweeteners in food production, e.g., ice cream [8] and analyzing the acceptability of such modified foods by consumers [9]. Some works describing the results of several investigations on the impact of artificial sweeteners on our environment have been published recently. These works concern the determination of new contaminants in surface and ground waters of rivers [10] and a few newly developed methods enabling the accurate identification and determination of these pollutants [11], as well as some technologies that could be used for the effective degradation [12] of the mentioned contaminants occurring in wastewaters and natural waters due to people’s daily lifestyle habits.

All kinds of sweeteners have been analyzed. Most of the reported investigations point to the separation, identification, and determination of sweetening agents in commercially available food products [13,14,15]. There are many reports on the determination of elements in natural sweeteners, sometimes in the context of nutritional profile assessment, such as sugar [16,17,18], honey [18,19], maple syrup [20], agave syrup [21], cane syrup [22], and nipa palm syrup [23], whereas only a few studies exist on the quantification of mineral constituents in artificial sweeteners, for example, aspartame [24,25], sodium cyclamate, sodium saccharin, sucralose, and acesulfame potassium [25]. The largest number of reported investigations implicate only a few elements, usually recognized as essential, such as Ca, Fe, K, Mg, Mn, Na, and Zn [16,17,20,21,22,25]. Only limited research papers have been devoted to multielement analyses [18,19,23,24]. Measurements based on the quantitative determination of major and trace elements have been performed mostly using spectroscopic methods, for example, flame photometry [25], flame atomic absorption spectrometry (FAAS) [16,17,22], inductively coupled plasma–optical emission spectroscopy (ICP-OES) [19,20,23,24], inductively coupled plasma–mass spectroscopy (ICP-MS) [19,24], and instrumental neutron activation analysis (INAA) [18]. Before analysis, samples were acid-digested in opened [24] or closed vessels [19] with the aid of microwaves, dissolved in water [20] or water preconcentrated with Dovex resins [16], or metals of interest were extracted using diluted acids from the matrix with the assistance of ultrasound energy [17,22].

The main aim of the present study was to determine and verify the identity of the main and trace elements in commercially available sweetening products, namely natural, plant-derived, and artificial sweeteners. The results of our study allow for the evaluation of these popular, commercially available products, which is a necessary step in the process of the verification of food identity, quality, and safety, which may be of interest to scientists such as analysts, toxicologists, or nutritionists, as well as consumers.

## 2. Results and Discussion

### 2.1. Identity of Sweeteners—Phase Analysis

**Figure 1:**
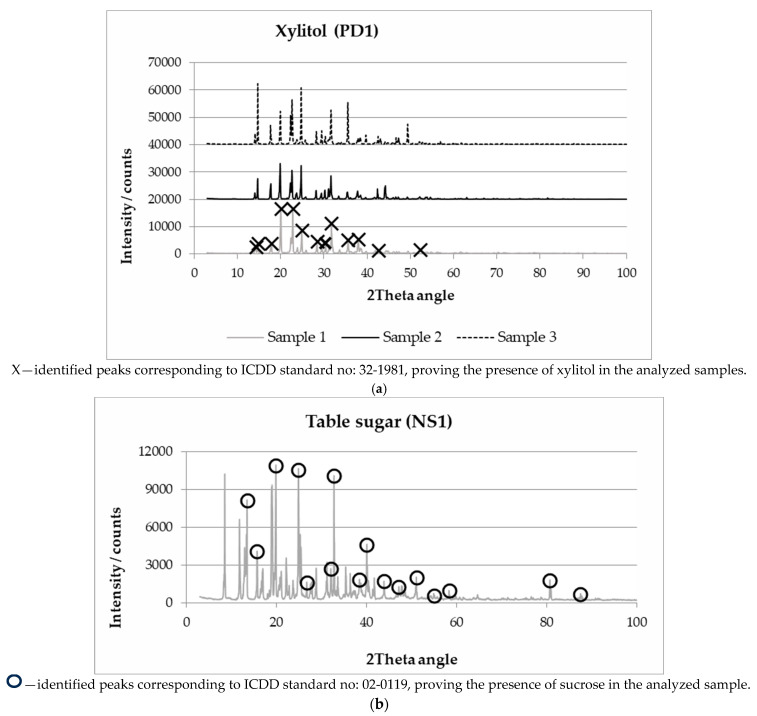
The experimental X-ray diffraction (XRD) patterns for (**a**) xylitol (PDS1) and (**b**) table sugar (NS1), where X—xylitol; **O**—sucrose.

Although the X-ray powder diffraction method has been frequently used to study the phase composition of various food products and their components, to the best of our knowledge, its application to investigate and confirm the composition and identity of sweeteners has not been reported to date. In the present work, the phase analysis of natural sweeteners, plant-derived sweeteners, artificial sweeteners, and composite sweetening products was carried out. A detailed description of the analyzed sweeteners is shown in Table 5 in the Section 3. The comparison of the measured diffraction peak positions and intensities with the reference patterns of crystalline materials allows us to confirm the identity of the analyzed products. Experimental diffractograms of xylitol and table sugar samples, showing the peaks whose position agrees with the peaks of the reference pattern, are shown in Figure 1a,b.

For the analyzed xylitol samples (PDS1), a sweetener derived, for example, from birch wood (genus: *Betula*, family: *Betulaceae*), the sweetener’s identity was confirmed by the clear agreement of the position of the experimental diffraction peaks and the standard (ICDD standard no: 32-1981) situated at 2-theta angles: 14.0, 14.6, 17.7, 20.0, 22.6, 24.6, 28.1, 30.2, 31.4, 35.6, 37.8, 42.3, and 53.4° (indicated with x in the diffractogram in Figure 1a). The verified peaks’ shape, i.e., their intensity and area, slightly differed from sample to sample, but those were the criteria used for the analysis of the constituent crystallographic structure compatibility. For all the analyzed one-component sweeteners, i.e., plant-derived and artificial, it was found that the recorded diffraction peaks were distinctly associated with the presence of the sweetening agent, which allowed us to confirm the authenticity of the product. For example, in the experimental diffractograms of aspartame or sodium cyclamate, no additional peaks indicating the presence of fillers or impurities were observed. In the case of composite samples and table sugar, i.e., the product of natural origin, industrially extracted from sugar beet, the acquired diffractograms were more complex. However, in their case, too, it was possible to unambiguously identify the sweetener by using the standards available in the ICDD PDF-2 database. As can be seen in the experimental diffractogram of sugar (NS1) (shown in Figure 1b), the confirmation of the presence of sucrose in the product was possible. According to the ICDD PDF-2 base (standard no: 02-0119), the diffraction peaks at 13.5, 15.7, 19.9, 24.9, 26.8, 32.1, 32,8, 38.4, 40.1, 43.9, 47.2, 51.1, 55.0, 58.4, 80.8, and 87.6° (2θ) were related to the presence of sucrose in the analyzed table sugar sample. In addition to the peaks indicating the presence of sucrose, several additional ones were acquired that might be due to the presence of other crystallographic alternatives of the compound of interest or additives and impurities. Unfortunately, in the case of the sugar analysis, the definite identification of additional constituents was not possible with the available reference patterns base version (PDF-2).

### 2.2. Concentrations of Macroelements, Microelements, and Trace Elements

The results of the total concentrations of elements determined in all the analyzed sweeteners (natural sweeteners, plant-derived sweeteners, artificial sweeteners, and composite sweetening products) using ICP-OES are shown in Table 1. They are expressed in µg·g^−1^ as the arithmetic mean value (*n* = 3) along with their standard deviations. A detailed description of the analyzed samples is given in Table 5 in the Section 3.

In all the analyzed sweeteners, the total concentrations of Al, B, Ba, Fe, Mn, Sr, and Zn were quantified. Ag and Mo were determined only in table sugar (S1). Bismuth and V were determined in two of the products, i.e., sugar (S1) and the two-component sample no. 1 (CS1) (Bi) as well as sugar (S1) and potassium acesulfame (AS2) (V). The smallest number of elements was quantified in the samples AS3, AS2, and PDS3, i.e., one-component sweeteners, comprising two artificial and one plant-derived. In contrast, the largest number, but not all, of the elements was determined in samples that were not one-component products: CS1 and CS2. The Mg content ranged from 0.04 µg·g^−1^ in the S1 sample to 490 µg·g^−1^ in the PDS2 sample. The variation in the Mg concentrations determined in the examined samples reached five orders of magnitude. The highest concentrations of Mg (490 and 141 µg·g^−1^) were determined in PDS2 (erythritol) and CS1 (erythritol + steviol glycosides) samples. In CS2, containing magnesium stearate in its composition, the Mg content was 75 µg·g^−1^. The concentration range of two orders of magnitude was observed for the concentrations of B, Fe, and Pb. The observed differences were more than fifty times for Al, Cu, Mn, and Sr. The content of Ca, the only element quantified in all sweeteners except table sugar (S1), ranged between 1.9 (AS2) and 40 (AS3) µg·g^−1^. Among all the examined elements determined in all the analyzed samples, the highest concentration was noted for Fe. The content of this element ranged from 0.26 (S1) to 28 (AS4) µg·g^−1^. A relatively high content, compared with all the analyzed samples, was also observed for Zn. The concentration of this element was between 0.14 (AS2) and 1.73 (AS3) µg·g^−1^. Except for the samples PDS3 (sorbitol) and S1 (table sugar), contents lower than 2.0 µg·g^−1^ were determined for Al, B, Ba, Cu, Co, and Mn. In the case of the aforementioned samples, the B concentration in PDS3 (sorbitol) was 4.2 µg·g^−1^, while the Cu content in S1 (table sugar) equaled 3.5 µg·g^−1^. Finally, such elements as Sr and Ti were found to be less than 0.5 µg·g^−1^ in the examined samples.

Taking into account the range of the element content depending on the type of sweetener, it was concluded that the PDS3 (sorbitol) and S1 (table sugar) samples had the smallest differences, while the largest differences were characteristic for PDS2 (erythritol), i.e., the one in which the highest Mg concentration was determined. The content of the elements determined in artificial sweeteners (AS1–AS4) varied from about five hundred times (AS2) to more than two thousand times (AS4), depending on their type.

Considering the content of metals that are toxic and can be harmful to humans, e.g., Cd, Cr, Ni, and Pb, it is clear that their presence in food products is undesirable and worrying, even if is detected in food additives that are generally consumed in small quantities. Cadmium was only present in the two multicomponent samples (CS1 and CS2) at concentrations of 0.02 and 0.01 μg·g^−1^, respectively. Chromium was present in all the analyzed samples except for the samples PDS1 and AS3; its concentration ranged from 0.04 (PDS2, erythritol) to 0.41 µg·g^−1^ (AS1, aspartame). The concentration of Ni changed from 0.45 (CS1) to 1.10 (PDS2) µg·g^−1^. The content of Pb was within the range of 0.02 (S1, table sugar)–3.5 µg·g^−1^ (AS4, sodium saccharin). Table 2 provides the data on the potential intake of toxic elements related to the consumption of a portion equivalent to two flat teaspoons (sugar and plant-derived sweeteners) as well as the amount allowed for daily consumption by a man with an average body mass, i.e., 70 kg (artificial sweeteners).

The content of the toxic elements determined in the analyzed sweeteners was compared with the actual limits set by the WHO and the EU for drinking water and the maximal level of metals for foodstuffs given by the European Union. As can be seen in Table 1, the estimated levels of toxic elements in sweeteners significantly exceed the quantities permitted for drinking water. Cadmium can enter the organism through the ingestion of composite products. Its content was not higher than the range set for the different foodstuffs. Chromium and lead were present in all types of the analyzed sweeteners. The consumption of two teaspoons of table sugar, plant-derived sweeteners, and complex products or the permitted daily dose of artificial sweeteners causes a significant amount of Cr and Pb to enter the human organism, in the case of Pb higher than the maximal value allowed for foods. The amount of Ni introduced through the consumption of sweeteners exceeded from almost one hundred to over five hundred times the standards set for drinking water.

### 2.3. Statistical Analysis

ANOVA was used to test for statistical differences between the means of elements obtained for the analyzed samples. The results of the statistical evaluation of the mean concentrations of elements obtained for the table sugar (natural sweetener, S1), plant-derived sweeteners (xylitol, PDS1; sorbitol, PDS2; and erythritol, PDS3), artificial sweeteners (aspartame, AS1; potassium acesulfame, AS2; sodium cyclamate, AS3; and sodium saccharin, AS4), and composed products (CP-1 containing erythritol + steviol glycosides and CP2 containing sorbitol + aspartame + magnesium stearate) are shown in Table 3.

According to the ANOVA test results, statistically significant differences were observed between the mean concentrations of Al, B, Ba, Fe, Mn, Sr, and Zn in the different sweetener samples, i.e., elements that were quantified in all the analyzed samples. Significant differences in the concentrations of Bi and V (both determined only in two samples) were also observed. The contents of Ba, Ca, Mn, Sr, and Zn, determined in AS3, varied significantly compared with the corresponding values determined in the samples of artificial sweeteners (AS1, AS2, and AS4), as well as table sugar (S1), plant-derived sweeteners (PDS1-PDS3), and samples of composed products (CP1 and CP2). Statistically significant differences in the element content were also observed for artificial sweeteners, i.e., AS4 (Co, Fe, Mn, and Pb), AS3 (Ba, Ca, and Mn), AS1 (Cr and Fe), and AS2 (Ca). The ANOVA results revealed statistically significant differences between the Al concentration determined in the samples S1 and PDS2 and the content of this element in other sweeteners. The boron content in sample PDS3 differed significantly from that observed in samples S1, PDS1, PDS2, and AS1. Also, the Mg content measured in PSD2 (erythritol), as well as CP1 and CP2, i.e., the complex samples containing erythritol, were significantly different from the values noted for the rest of the analyzed sweeteners.

### 2.4. Inter-Element Relationships

The potential relationships between the concentrations of elements determined in the analyzed sweeteners were also investigated. The Pearson correlation coefficients (*r*) were calculated considering both the type of sweetener and the kind of element. The elements’ concentration correlation matrix for the type of sweetener is shown in Table 4. Very strong and strong relationships are indicated as underlined italic fonts.

Based on these results, a very strong positive relationship (│*r*│ > 0.9) was observed for PDS1-AS3, PDS2-CP1, PDS2-CP2, AS1-AS4, and CP1-CP2 pairs of sweeteners. A strong correlation (0.7 < │*r*│ > 0.9) was found for the following pairs: PDS1-AS2, PDS2-AS2, PDS3-AS2, AS2-AS3, AS2-CP1, and AS2-CP2. A moderate correlation (│*r*│ within the range of 0.4–0.7) was observed for PDS1-PDS3, PDS3-AS3, PDS3-AS4, and AS2-AS4. No relationship or almost negligible correlations were found for the rest of the examined pairs of sweeteners. Among the observed associations, both positive and negative inter-relationships were observed. However, it must be noted that all the very strong, strong, and moderate relations were only positive, while the coefficients (*r*) indicating weak or no relationship were negative and positive.

Considering the level of elements determined in the different sweeteners, a very strong positive relationship (r > 0.9) was observed for Co-Cr, Co-Fe, Co-Mn, Co-Pb, and Mn-Pb pairs of elements. A strong (0.7 < │*r*│ > 0.9) positive relationship between the elements was found for Al-Ti, B-Co, B-Pb, Ba-Ni, Ba-Zn, Ca-Zn, Cr-Fe, Fe-Mn, Fe-Pb, Mg-Ni, Mg-Sr, and Ni-Sr. In contrast, a strong negative inter-relationship was only found for Co-Ti. A moderate correlation (0.4 < │*r*│ > 0.7) was found for Al-Co, Al-Cu, Al-Fe, Al-Mn, Al-Ni, Al-Pb, B-Cu, B-Ni, Ba-Ca, Ba-Cu, Ba-Sr, Ca-Co, Ca-Sr, Cr-Mg, Cr-Mn, Cr-Pb, Cu-Zn, Pb-Ti, Pb-Zn, and Sr-Zn. Considering both positive and negative associations, no or almost negligible association was observed for the rest of the examined elements.

## 3. Materials and Methods

### 3.1. Samples and Reagents

Ten kinds of commercially available sweeteners, both natural and artificial, typically used as food additives, were chosen for investigation. These included one-component as well as complex products. The description of examined sweeteners is given in Table 5. All sweetener samples were purchased in Wrocław (Poland). Before analysis, they were ground to a fine powder using an agate mortar and pestle.

All experiments were carried out with the use of high-purity deionized water (18.3 MΩ cm^−1^) obtained from an EASYpure™ system (Barnstead, Thermolyne Corporation, Dubuque, IA, USA). All the chemicals used in this study were at least of analytical grade and were verified for possible contamination.

Before use, all glassware and plastic bottles were washed with distilled water, cleaned with diluted nitric acid in an ultrasonic bath, rinsed several times with deionized water, and dried.

### 3.2. Microwave-Assisted Closed-Vessel Wet Digestion

Accurately weighed portions of samples (about 0.5 g) were transferred into the Teflon digestion vessels. Next, 5.0 mL of concentrated nitric acid (65% (*m*/*v*) HNO_3_, Suprapur, Merck KGaA, Darmstadt, Germany) was added and kept overnight for predigestion. The next day, 1.0 mL of 30% (*m*/*v*) H_2_O_2_ (Merck KGaA, Darmstadt, Germany) was added to each vial. The decomposition of the predigested samples was carried out in a microwave digestion system (Milestone, Apeldoorn, The Netherlands, MLS-1200, MEGA), using a six-step program with a maximum power of 600 W. After finishing the digestion program and cooling the vessels, they were opened and the clear and colorless digests were quantitatively transferred to the 25.0 mL volumetric flasks and finally brought up to the volume with deionized water. The resulting sample solutions were stored at 4 °C prior to the analysis.

Three parallel samples (*n* = 3) of each examined sweetener were prepared and analyzed. With each set of digested samples, the blank samples were simultaneously prepared by running through the complete sample preparation procedure, analyzed, and used for the correction of the final results of the multielement analysis using ICP-OES.

### 3.3. Measurements

An X-ray powder diffraction method (XRD) was used to investigate the identity and pureness of natural and artificial sweeteners. Measurements were performed in a symmetric θ/2θ Bragg–Brentano geometry using a Philips X’PERT system. The diffractometer was equipped with a CuKα (λ = 0.154 nm) source, vertical goniometer, and angle and position reflex registration counters. The measuring range of the 2-theta angle (2θ) was 3–100°, step—0.02°, a single-pulse counting time—2 s, voltage—40 kV, and current—30 mA. The identification of the phases was accomplished by comparing the experimental powder diffraction patterns with reference patterns collected in the Powder Diffraction Files (International Centre for Diffraction Data PDF-2 base).

Measurements of the total concentrations of the studied elements, i.e., Ag, Al, B, Ba, Bi, Ca, Cd, Co, Cr, Cu, Fe, Mg, Mn, Mo, Ni, Pb, Sr, Ti, V, and Zn, were taken using an Agilent bench-top optical emission spectrometer (model 720) with an axially viewed Ar-ICP (Agilent Technologies Inc., Santa Clara, CA, USA). The spectrometer operated under the following settings: the RF power—1200 W; Ar flow rates—15.0 (plasma); 1.5 (auxiliary) and 0.75 dm^3^∙min^−1^ (nebulizing); the sample flow rate—0.75 dm^3^∙min^−1^; instrument stabilization and sample uptake delays—15 s; rinse time—10 s; replicate (*n* = 3); and read time—1 s. The intensities of the emission lines (in nm) were 328.068 (Ag), 396.152 (Al), 249.678 (B), 455.403 (Ba), 223.061 (Bi), 422.673 (Ca), 226.502 (Cd), 238.892 (Co), 267.716 (Cr), 324.754 (Cu), 238.204 (Fe), 285.213 (Mg), 257.610 (Mn), 202.032 (Mo), 231.604 (Ni), 220.353 (Pb), 407.771 (Sr), 336.122 (Ti), 292.401 (V), and 213.857 (Zn). Background-corrected net intensities of these lines were used in this study. A fitted background mode with seven points per line profile was applied for background correction. Five-point external calibration curves in the range of 0.25–5.0 mg∙dm^−3^ were used to quantify all the studied elements. Standard solutions such as ICP multielement standard solution no. IV and CertiPUR single standard solutions of Mo, P, Ti, and V (all provided by Merck) were used to prepare calibration standards.

The precision of measurements of the analyzed sweetener samples was good. For most of the examined elements determined at the ppm (μg·g^−1^) level, the relative standard deviations (RSDs) calculated for the individual samples were lower than 10%. Only in the case of elements determined in very low levels (of the order of ng·g^−1^) were the RSD values higher, i.e., up to 30% and more. Nevertheless, it was acceptable and predictable for such low quantities of elements present.

Due to the absence of a certified reference material (CRM) with a composition similar to the analyzed sweeteners, the validity of the results of the multielement analysis obtained using ICP-OES was verified with spike-and-recovery experiments using the standard addition method. Two different concentrations of elements in the final sample solutions (0.5 and 1.0 μg·mL^−1^) were tested, which were added to the selected samples before their wet digestion. The concentration levels of the additions depended on the concentrations of elements determined in the test sample. The assessed recovery ranged from 76.6% to 102.2%, thus verifying the validity of the spectrometric determination.

### 3.4. Statistical Analyses

One-way analysis of variance (ANOVA) was used to test for statistical differences between the means of elements obtained for the examined sweeteners. The least significant difference and Tukey–Kramer post hoc multiple-comparison tests were performed for the verification of which of the elements’ concentration means were significantly different from each other. The differences between the mean concentration values were considered significant at *p* < 0.05. All the statistical tests were performed using Data Analysis Toolpak in Excel.

## 4. Conclusions

For the first time, different kinds of commercially available sweetening products, i.e., natural, plant-derived, and artificial sweeteners, were analyzed in terms of their authenticity and element content, using XRD and ICP-OES methods, respectively.

Our investigations proved the identity of all the analyzed sweeteners; it is clear that all the products contained the expected sweetening agent. The X-ray powder diffraction method is a useful tool and suitable for this type of analysis; moreover, as a non-destructive method, it can be used before other steps of the analytical procedure.

In general, the concentrations of macroelements and microelements considerably differed depending on the type of sweetener. However, despite the statistically significant differences in the content of elements and unequivocal correlations between elements, it was not possible to identify the elements whose content would allow for the assignment of a sweetener to a specific group (natural, plant-derived, or artificial). According to our analysis, it can be concluded that, except for Ca and Mg, these sweeteners are poor sources of nutrient elements. However, and importantly, the examined sweeteners contain toxic elements like Cd, Cr, Ni, and Pb at quantities significantly exceeding those permitted for drinking water. Hence, their daily consumption may cause health problems, which clearly confirms the need for the control of sweeteners’ mineral composition to ensure consumer protection.

There are two major limitations in this study that could be addressed in future research to improve the generalizability of the results. The first is the relatively small sample size and the fact that the sampling was limited to the sweetening product available in Wrocław. The second limitation is the application of only one method of element quantification. Future studies should engage the analysis of a larger number of the same kind of sweetener samples (different producers or altered sampling locations) and the use for example ICP-MS method for the quantification of metals, especially present in samples at very low quantities, which provide complementary results.

## Figures and Tables

**Table 1:** The total concentrations of the studied elements in sweeteners (in µg·g^−1^).

Product Element	S1	PDS1	PDS2	PDS3	AS1	AS2	AS3	AS4	CP1	CP2
Ag	0.42 ± 0.05	<*5 ng·g*^−1 a^	<*5 ng·g*^−1 a^	<*5 ng·g*^−1 a^	<*5 ng·g*^−1 a^	<*5 ng·g*^−1 a^	<*5 ng·g*^−1 a^	<*5 ng·g*^−1 a^	<*5 ng·g*^−1 a^	<*5 ng·g*^−1 a^
Al	1.80 ± 0.05	0.9 ± 0.1	1.5 ± 0.2	1.2 ± 0.1	1.0 ± 0.1	0.26 ± 0.03	0.31 ± 0.01	0.03 ± 0.01	0.66 ± 0.05	0.69 ± 0.04
B	0.02 ± 0.00	1.0 ± 0.1	1.5 ± 0.1	4.2 ± 0.6	0.9 ± 0.4	0.87 ± 0.04	1.2 ± 0.3	<*0.3 µg/dm*^3^	1.13 ± 0.04	1.12 ± 0.04
Ba	0.61 ± 0.03	0.05 ± 0.02	0.52 ± 0.03	0.06 ± 0.01	0.05 ± 0.01	0.05 ± 0.00	1.4 ± 0.3	0.14 ± 0.01	0.13 ± 0.02	0.07 ± 0.01
Bi	4 ± 1	<*3 ng·g*^−1 a^	<*3 ng·g*^−1 a^	<*3 ng·g*^−1 a^	<*3 ng·g*^−1 a^	<*3 ng·g*^−1 a^	<*3 ng·g*^−1 a^	<*3 ng·g*^−1 a^	0.5± 0.1	<*3 ng·g*^−1 a^
Ca	<*0.01 ng·g*^−1 a^	13 ± 4	15 ± 3	3.1 ± 0.4	7.0± 0.4	1.9 ± 0.6	40 ± 10	10.1 ± 0.8	30.2 ± 0.8	26 ± 2
Cd	<*0.4 ng·g*^−1 a^	<*0.4 ng·g*^−1 a^	<*0.4 ng·g*^−1 a^	<*0.4 ng·g*^−1 a^	<*0.4 ng·g*^−1 a^	<*0.4 ng·g*^−1 a^	<*0.4 ng·g*^−1 a^	<*0.4 ng·g*^−1 a^	0.02 ± 0.00	0.01 ± 0.01
Co	0.03 ± 0.01	0.02 ± 0.02	0.13 ± 0.03	<*0.8 ng·g*^−1 a^	<*0.8 ng·g*^−1 a^	<*0.8 ng·g*^−1 a^	0.11 ± 0.02	0.99 ± 0.03	<*0.8 ng·g*^−1 a^	0.10 ± 0.01
Cr	0.08 ± 0.02	<*0.5 ng·g*^−1 a^	0.04 ± 0.01	0.18 ± 0.03	0.41 ± 0.01	0.02 ± 0.00	<*0.5 ng·g*^−1 a^	0.3 ± 0.2	0.09 ± 0.01	0.04 ± 0.01
Cu	3.5 ± 1.1	0.06 ± 0.01	0.62 ± 0.01	0.06 ± 0.01	1.63 ± 0.05	0.13 ± 0.00	<*0.6 ng·g*^−1 a^	0.10 ± 0.01	0.20 ± 0.01	0.47 ± 0.09
Fe	0.26 ± 0.01	1.3 ± 0.2	1.6 ± 0.5	1.8 ± 0.2	18 ± 2	0.85 ± 0.09	2 ± 1	28 ± 7	3.0 ± 0.4	1.7 ± 0.3
Mg	0.04 ± 0.01	0.60 ± 0.08	490 ± 20	0.7 ± 0.1	0.7 ± 0.1	<*0.01 ng·g*^−1 a^	2.69 ± 0.03	1.8 ± 0.7	141 ± 3	75 ± 1
Mn	0.04 ± 0.01	0.04 ± 0.01	0.05 ± 0.01	0.04 ± 0.01	0.06 ± 0.01	0.02 ± 0.00	0.57 ± 0.04	1.6 ± 0.1	0.09 ± 0.01	0.05 ± 0.01
Mo	0.39 ± 0.06	<*0.8 ng·g*^−1 a^	<*0.8 ng·g*^−1 a^	<*0.8 ng·g*^−1 a^	<*0.8 ng·g*^−1 a^	<*0.8 ng·g*^−1 a^	<*0.8 ng·g*^−1 a^	<*0.8 ng·g*^−1 a^	<*0.8 ng·g*^−1 a^	<*0.8 ng·g*^−1 a^
Ni	<*1.3 ng·g*^−1 a^	0.6 ± 0.1	1.1 ± 0.3	0.17 ± 0.08	0.61 ± 0.09	<*1.3 ng·g*^−1 a^	<*1.3 ng·g*^−1 a^	0.55 ± 0.06	0.45 ± 0.06	0.6 ± 0.1
Pb	0.02 ± 0.00	0.19 ± 0.01	<*3 ng·g*^−1 a^	<*3 ng·g*^−1 a^	0.48 ± 0.00	0.3 ± 0.1	<*3 ng·g*^−1 a^	3.5 ± 0.2	0.3 ± 0.1	0.38 ± 0.03
Sr	0.05 ± 0.01	0.02 ± 0.00	0.30 ± 0.02	0.02 ± 0.01	0.03 ± 0.00	0.01 ± 0.00	0.22 ± 0.04	0.01 ± 0.00	0.09 ± 0.00	0.08 ± 0.00
Ti	<*0.13 ng·g*^−1 a^	0.09 ± 0.01	0.08 ± 0.01	0.09 ± 0.01	0.13 ± 0.01	0.02 ± 0.00	0.04 ± 0.01	0.02 ± 0.00	0.08 ± 0.01	0.06 ± 0.01
V	0.17 ± 0.04	<*0.4 ng·g*^−1 a^	<*0.4 ng·g*^−1 a^	<*0.4 ng·g*^−1 a^	<*0.4 ng·g*^−1 a^	0.02 ± 0.00	<*0.4 ng·g*^−1 a^	<*0.4 ng·g*^−1 a^	<*0.4 ng·g*^−1 a^	<*0.4 ng·g*^−1 a^
Zn	0.5 ± 0.1	0.5 ± 0.1	0.26 ± 0.09	0.31 ± 0.06	0.6 ± 0.1	0.14 ± 0.03	1.73 ± 0.04	0.23 ± 0.01	0.42 ± 0.03	0.37 ± 0.02

S1—natural sweetener (table sugar); PDS1–PDS3—plant-derived sweeteners (xylitol, sorbitol, and erythritol); AS1–AS4—artificial sweeteners (aspartame, potassium acesulfame, sodium cyclamate, and sodium saccharin); CP1–CP2—composite products (erythritol + steviol glycosides and sorbitol + aspartame + magnesium stearate); ^a^ below the limit of quantification (LOQ).

**Table 2:** The toxic elements’ potential intake caused by the sweetener consumption.

Element	Content of Element (μg) in Sweetener Portion				Permissible Limits for Drinking Water According to EU [26] and WHO [27]	Maximum Level of Metal in Foodstuffs [28] (μg/g)
	S1 Portion: 10 g	PDS1–PDS4 Portion: 14 g for PDS1 or 10 g	AS1–AS4 AS1: 2.8 g; AS2: 0.63 g; AS3: 0.49 g; AS4: 0.35 g	CP1–CP2 Portion: 10 g		
Cd	nd ^a^	nd ^a^	nd ^a^	0.04–0.22	0.005 mg/dm^3^	0.05 (meat, mussel meat of fish and vegetables)–1.0 (kidney, Bivalve mollusks, Cephalopods)
Cr	0.79	0.42–1.8	0.01–1.1	0.11–0.88	0.05 mg/dm^3^	value not given
Ni	nd ^a^	1.7–11	1.7	1.6–4.5	0.02 mg/dm^3^	value not given
Pb	0.15	2.7	0.17–1.3	1.1–3.4	0.01 mg/dm^3^	0.020 (raw milk and infant formulae)–1.5 (Bivalve mollusks)

S1—natural sweetener (table sugar); PDS1–PDS3—plant-derived sweeteners (xylitol, sorbitol, and erythritol); AS1–AS4—artificial sweeteners (aspartame, potassium acesulfame, sodium cyclamate, and sodium saccharin); CP1–CP2—composite products (erythritol + steviol glycosides and sorbitol + aspartame + magnesium stearate); ^a^ nd—not determined.

**Table 3:** The pairs of sweeteners for which statistically significant differences were discovered.

Element	The Pairs of Sweeteners	Element	The Pairs of Sweeteners
Al	S1-PDS1, S1-AS3, S1-AS4, S1-CP1, S1-CP2; PDS2-AS2, PDS2-AS3, PDS2-AS4;	Mg	S1-PDS2, S1-CP1, S1-CP2; PDS1-PDS2, PDS1-CP1, PDS1-CP2; PDS2-AS1, PDS2-AS3, PDS2-AS4, PDS2-CP1, PDS2-CP2; PDS3-PDS2, PDS3-CP1, PDS3-CP2; AS3-CP1, AS3-CP2; AS4-CP1, AS4-CP2; CP1-CP2;
B	S1-PDS3; PDS1-PDS3; PDS3-PDS2, PDS3-AS1;	Mn	S1-AS3, S1-AS4; PDS1-AS4; PDS2-AS4; PDS3-AS3; AS1-AS4; AS2-AS3, AS2-AS4; AS3-AS4; AS3-CP1, AS3-CP2; AS4-CP1, AS4-CP2;
Ba	S1-PDS1, S1-AS1, S1-AS3; PDS2-AS1, PDS2-AS3, PDS2-CP2; AS1-AS3; AS2-AS3; AS3-AS4, AS3-CP1, AS3-CP2;	Ni	PDS2-PDS3;
Bi	S1-CP1;	Pb	S1-AS4; PDS1-AS4; PDS3-AS4; AS1-AS4; AS2-AS4; AS4-CP1, AS4-CP2;
Ca	AS1-AS3; AS2-AS3, AS2-CP1, AS2-CP2; AS3-AS4,	Sr	S1-PDS3, S1-AS3; PDS1-PDS3; PDS2-AS1, PDS2-AS2, PDS2-AS4, PDS2-CP1, PDS2-CP2; PDS3-PDS2, PDS3-AS3; AS1-AS3; AS2-AS3; AS3-AS4; AS3-CP1, AS3-CP2;
Co	S1-AS4; PDS1-AS4; PDS2-AS4, AS3-AS4,	V	S1-AS2;
Cr	S1-AS1; PDS2-AS1; PDS3-AS4; AS1-CP1, AS1-CP2;	Zn	S1-AS3; PDS2-AS3; PDS3-AS3; AS1-AS3; AS2-AS3; AS3-AS4; AS3-CP1, AS3-CP2;
Fe	S1-AS1, S1-AS4; PDS1-AS1, PDS1-AS4; PDS2-AS1, PDS2-AS4; PDS3-AS1, PDS3-AS4; AS1-AS2, AS1-AS3, AS1-CP1, AS1-CP2; AS2-AS4; AS3-AS4; AS4-CP1, AS4-CP2;		

S1—natural sweetener (table sugar); PDS1–PDS3—plant-derived sweeteners (xylitol, sorbitol, and erythritol); AS1–AS4—artificial sweeteners (aspartame, potassium acesulfame, sodium cyclamate, and sodium saccharin); CP1–CP2—composite products (erythritol + steviol glycosides and sorbitol + aspartame + magnesium stearate).

**Table 4:** Pearson correlation coefficient (*r*) matrix for the different sweeteners.

	S1	PDS1	PDS2	PDS3	AS1	AS2	AS3	AS4	CP1	CP2
S1	1									
PDS1	−0.073	1								
PDS2	−0.180	−0.038	1							
PDS3	−0.203	0.558	−0.024	1						
AS1	−0.041	0.355	−0.090	0.384	1					
AS2	−0.118	*0.892*	*0.896*	*0.832*	0.577	1				
AS3	−0.206	*0.995*	−0.025	0.481	0.290	*0.855*	1			
AS4	−0.169	0.328	−0.051	0.637	*0.986*	0.608	0.265	1		
CP1	−0.195	0.142	*0.983*	0.072	−0.012	*0.879*	0.143	0.004	1	
CP2	−0.160	0.274	*0.953*	0.138	0.025	*0.872*	0.282	0.045	*0.992*	1

S1—natural sweetener; PDS1–PDS3—plant-derived sweeteners; AS1–AS4—artificial sweeteners; CP1–CP22—complex products.

**Table 5:** Characteristics of the examined sweeteners.

Sample	Product	Common Name or European Code	Acceptable Daily Intake
S1	Table sugar	Sucrose distilled from Sugar beets	
PDS1	Xylitol	E967	quantum satis ^a^
PDS2	Sorbitol	E420	quantum satis ^a^
PDS3	Erythritol	E968	quantum satis ^a^
AS1	Aspartame	E951	40 mg/kg bw/day [29,30] 50 mg/kg bw/day [31]
AS2	Potassium acesulfame	E950	9 mg/kg bw/day [29] 15 mg/kg bw/day [30,31]
AS3	Sodium cyclamate	E952	7 mg/kg bw/day [29] 11 mg/kg bw/day [30] prohibited [31]
AS4	Sodium saccharin	E954	5 mg/kg bw/day [29,30] 15 mg/kg bw/day [31]
CP1	Erythritol (PDS3) + Steviol glycosides	E968 + E960	quantum satis ^a^
CP2	Sorbitol (PDS2) + Aspartame (AS1) + Magnesium stearate	E420 + E951 + E572	E951: 40 mg/kg bw [29,30] E420: quantum satis ^a^

S1—natural sweetener; PDS1–PDS3—plant-derived sweeteners; AS1–AS4—artificial sweeteners; CP1–CP22—composite products; ^a^ quantum satis means that no maximum level is specified; however, the added amount of this additive must not be higher than is necessary to achieve the intended purpose.

## Data Availability

The data presented in this study are available on request from the corresponding author.

## Sample Availability

Not available.
